# The vaginal microbiome is stable in prepubertal and sexually mature Ellegaard Göttingen Minipigs throughout an estrous cycle

**DOI:** 10.1186/s13567-015-0274-0

**Published:** 2015-10-28

**Authors:** Emma Lorenzen, Egle Kudirkiene, Nicole Gutman, Anette Blak Grossi, Jørgen Steen Agerholm, Karin Erneholm, Christina Skytte, Marlene Danner Dalgaard, Anders Miki Bojesen

**Affiliations:** Section for Veterinary Reproduction and Obstetrics, Department of Large Animal Sciences, Faculty of Health and Medical Sciences, University of Copenhagen, Copenhagen, Denmark; Chlamydia Vaccine Research, Department of Infectious Disease Immunology, Statens Serum Institut, Copenhagen, Denmark; Section for Veterinary Clinical Microbiology, Department of Veterinary Disease Biology, Faculty of Health and Medical Sciences, University of Copenhagen, Copenhagen, Denmark; Ellegaard Göttingen Minipigs A/S, Dalmose, Denmark; CiToxLab Scantox, Lille Skensved, Denmark; DTU Multi-Assay Core, Department of Systems Biology, Center for Biological Sequence Analysis, Technical University of Denmark, Lyngby, Denmark

## Abstract

Although the pig has been introduced as an advanced animal model of genital tract infections in women, almost no knowledge exists on the porcine vaginal microbiota, especially in barrier-raised Göttingen Minipigs. In women, the vaginal microbiota plays a crucial role for a healthy vaginal environment and the fate of sexually transmitted infections such as *Chlamydia trachomatis* infections. Therefore, knowledge on the vaginal microbiota is urgently needed for the minipig model. The aim of this study was to characterize the microbiota of the anterior vagina by 16 s rRNA gene sequencing in prepubertal and sexually mature Göttingen Minipigs during an estrous cycle. The dominating phyla in the vaginal microbiota consisted of *Firmicutes, Proteobacteria*, *Actinobacteria*, *Bacteriodetes* and *Tenericutes*. The most abundant bacterial families were *Enterobacteriaceae*, unclassified families from *Gammaproteobacteria*, *Clostridiales* Family XI Incertae Sedis, *Paenibacillaceae*, *Lactobacillaceae*, *Ruminococcaceae* and *Syntrophaceae*. We found a higher abundance of *Lactobacillaceae* in the prepubertal Göttingen Minipigs compared to sexually mature non-pregnant Göttingen Minipigs. However, correlation tests and diversity parameters revealed a very stable vaginal microbiota in the Göttingen Minipigs, both before and after sexual maturity and on different days throughout an estrous cycle. The vaginal microbiota in Göttingen Minipigs was not dominated by lactobacilli, as it is in women and according to our results the minipig vaginal microbiota is very stable, in opposite to women. These differences should be considered when using the minipig as a model of the genital tract in women.

**Electronic supplementary material:**

The online version of this article (doi:10.1186/s13567-015-0274-0) contains supplementary material, which is available to authorized users.

## Introduction

Trustworthy and predictive animal models are essential for gaining insight into diseases and new treatment strategies [1]. The female pig has been introduced as an advanced animal model of the genital tract in women [2–4] due to significant anatomical and physiological similarity between pigs and humans [5]. The pig has e.g. been introduced as a model of human genital *Chlamydia trachomatis* infection [2] in the need for an advanced animal model for evaluation of *C. trachomatis* vaccine candidates [6]. Göttingen Minipigs have been well characterised for use in biomedical research, and have benefits compared to conventional pigs, for example regarding the smaller size at sexual maturity, making them easy to handle, and they have a very strict and well-defined health status [7, 8]. Only very little is known about the porcine vaginal microenvironment and microbiota—and to our knowledge, nothing is known on the vaginal microbiota in barrier-bred Göttingen Minipigs.

In women, it is unclear exactly which underlying host-pathogen interactions and conditions that determine whether a sexually transmitted infection (STI) for example with *C. trachomatis* is cleared after an asymptomatic course or if it is allowed to ascend, become chronic and cause pathology in the Fallopian tubes [9]. It is known that an unbalanced vaginal microbiota in women increases the risk of an STI [9–11] and recent findings indicate that the host microbiota plays an important role for the outcome of an STI, including *C. trachomatis* infections [9, 11, 12]. The micro-environment is thought to exert its impact through direct inhibition of the infection of the epithelial cells, inhibition of proliferation and survival inside the epithelial cells and/or supporting faster clearance of the infected epithelial cells [12].

When determined by cultivation, the vaginal microbiota in women was predominantly composed of *Lactobacillus* spp., *Staphylococcus* spp., *Ureaplasma* spp., *Corynebacterium* spp., *Streptococcus* spp., *Peptostreptococcus* spp., *Gardnerella vaginalis*, *Bacteroides* spp., *Mycoplasma* spp., *Enterococcus* spp., *Escherichia coli*, *Veillonella* spp., *Bifidobacterium* spp. and *Candida* spp. [13, 14]. However, characterization of the microbiota in women by 16 s rRNA sequencing has revealed a very complex composition [15]. 16 s rRNA gene sequencing has shown that the microbiota is very susceptible to internal and external factors and that the composition can be highly fluctuating throughout a menstrual cycle [15, 16].

The porcine vaginal microbiota has so far, to our knowledge, only been determined by culturing. The vaginal microbiota in healthy conventional sows, detected by culture-based methods, includes a mixture of aerobic and anaerobic bacteria such as *Streptococcus* spp., *E. coli*, *Staphylococcus* spp., *Corynebacterium* spp., *Micrococcus* spp. and *Actinobacillus* spp. [17, 18]. Detection of microbiota based on culturing may only show a subpopulation of the microbiota, since non-culturable bacteria will not be detected by this method [19].

The very sparse knowledge on the vaginal microbiota in pigs, and the proposed great importance of the vaginal environment on the pathogenesis of vaginal infections, makes it urgently needed to gain knowledge on the porcine microbiota, when using the pig as a model of genital tract infections in women. Culture-independent methods are needed to achieve a comprehensive characterization of the porcine microbiota. The aim of this study was therefore to determine the composition of the vaginal bacterial microbiota by 16 s rRNA gene sequencing in prepubertal minipigs and sexually mature Göttingen Minipigs during an estrous cycle.

## Materials and methods

### Minipigs

The study was performed with ten prepubertal female Göttingen Minipigs (3-months-old) and ten sexually mature cycling, non-pregnant, and non-mated, female Göttingen Minipigs (approximately 12-months-old). Ellegaard Göttingen Minipigs A/S (EGM), Dalmose, Denmark, supplied all animals. The prepubertal minipigs were housed at the breeding facility while the sexually mature minipigs were moved to CiToxLab Scantox, Ejby, Denmark prior to sampling. At EGM the minipigs were housed under full barrier conditions, which include HEPA-filtered ventilation with 100% fresh air, overpressure, disinfection locks for diet and materials, shower locks for personnel and a closed manure system. Göttingen Minipigs do not share most of the common diseases found in conventional and Specific Pathogen Free (SPF) domestic swine. The minipigs are microbiologically defined and regularly tested for a number of pathogens according to the recommendations of the Federation of European Laboratory Animal Science Associations (FELASA) (for details, please refer to the supplier’s webpage [20]). At EGM, the animals were group housed in floor pens with approximately ten minipigs in each pen and with ionized straw as bedding. The animal room and pens were washed regularly and rinsed with water. The minipigs were fed on the pen floor with a SDS minipig diet [21] twice a day. The minipigs had enrichment material like metal chains and plastic balls in the pens and were allowed to run freely in the corridors and have nose-to-nose contact with animals in other pens once a day.

At CiToxLab Scantox the pens were cleaned and disinfected before arrival. The minipigs were single housed due to fighting, when initially group housed. They were stabled in concrete floor pens with sawdust (Jeluxyl, Jelu Werk GmbH, Josef Ehrler GmbH and Co KG, Ludwigsmühle, D-73494 Rosenberg, Germany) as bedding. The room was provided with filtered air at a temperature of 21 ± 3 °C. The temperature and relative humidity in the room was recorded hourly during the study. The ventilation system was designed to give 15 air changes per hour and the room was illuminated from 06:00 to 18:00 h to give a cycle of 12 h light and 12 h darkness.

The minipigs were fed an SDS minipig diet (SMP (E) SQC, Special Diets Services, Witham Essex, CM8 3AD, UK), twice daily in a bowl in an amount of approximately 250 g per animal per meal. The SDS diet was analysed for major nutritive components and relevant possible contaminants such as mesophilic spores, *Salmonellae*, *Enterobacteriaceae*, *Eschericia coli* and fungi. The minipigs were furthermore supplied with autoclaved grass seed straw from Hestehavegaard, Thomas Jørgensen, Ågerupvej 88, DK-4140 Borup, Denmark. Analyses for relevant possible contaminants were performed regularly (*E. coli*, *Enterobacteriaceae*, *Staphylococcus*, yeasts, moulds, *Clostridium* spp., *Bacillus cereus*, *Salmonella* spp., *Listeria monocytogenes*). They minipigs had 24 h access to domestic quality drinking water, regularly tested for possible contaminants (germ numbers, coliform bacteria and *E. coli*).

All minipigs were handled in accordance with the Danish animal experiments legislation and the study was approved by the Danish Animal Experiments Inspectorate, license number 2012-15-2934-00438.

The sexually mature minipigs were treated with altrenogest (Regumate^®^ Equine,MSD Animal Health, Ballerup, Denmark) (20 mg/minipig per day, orally for 18 days) to synchronize their estrous cycle. They were fed individually in this period to make sure that each minipig received a full daily dose. The minipigs went into clinical evident estrus 5 days after suspension of altrenogest (Regumate^®^) treatment (study day 0) and all minipigs entered clinical evident estrus again just before or on the last day of sampling (day 21).

### Vaginal swab sampling

**Figure 1:**
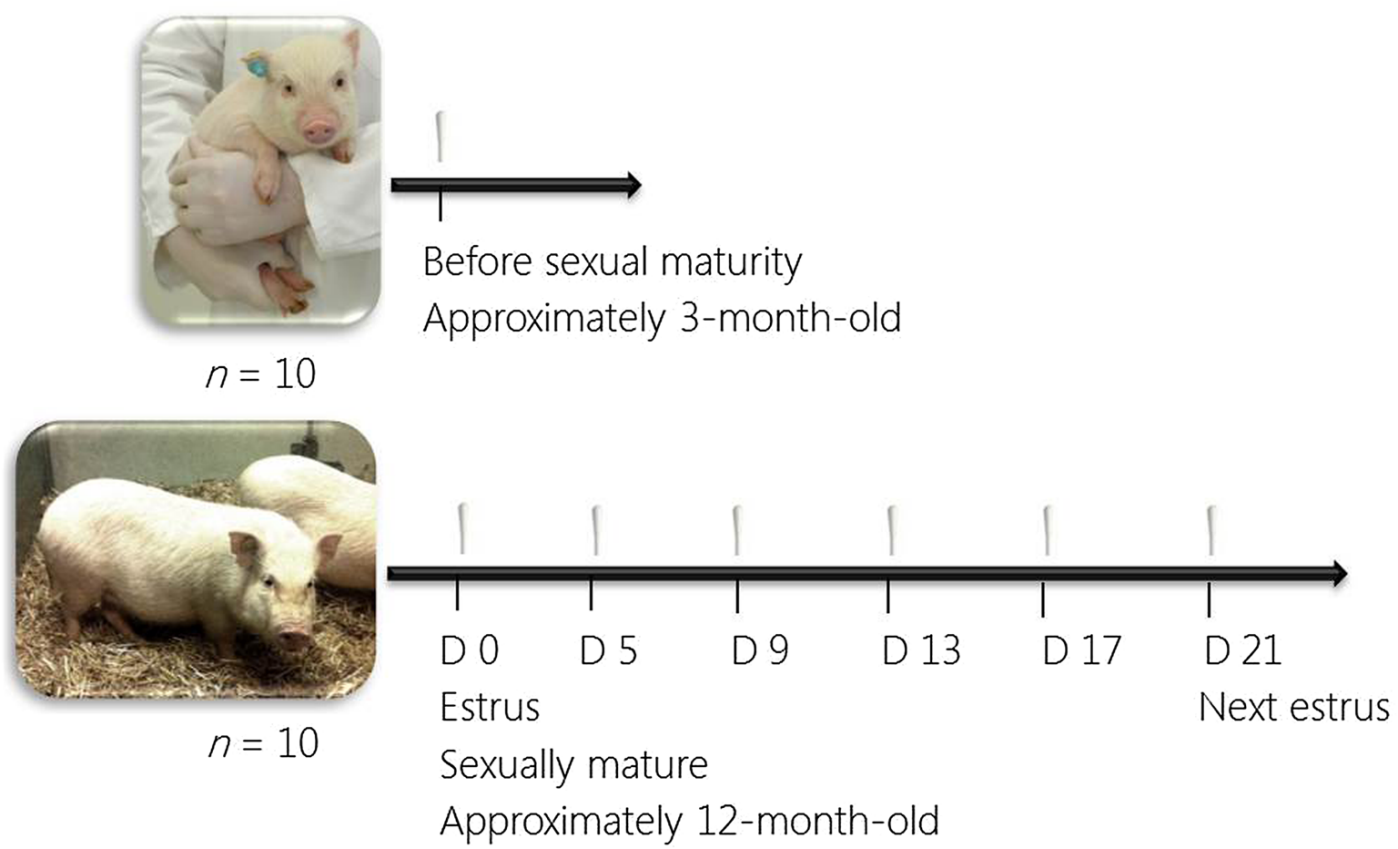
**Experimental setup.** The study was performed with two groups of Ellegaard Göttingen Minipigs; one group including ten prepubertal minipigs that were sampled one time and the other group including ten sexually mature non-pregnant minipigs that were hormonally synchronized and sampled six times during an estrous cycle. The first day of estrus after synchronization was designated day 0 and the following samples were taken at days 5, 9, 13, 17 and 21 in the estrous cycle.

Vaginal swabs were collected with a regular size Copan FLOQswab (Statens Serum Institut, Copenhagen, Denmark) at study days 0, 5, 9, 13, 17 and 21, i.e. during a full estrous cycle, for determination of the mucosal microbiota (Figure 1). Clean disposable gloves were used to avoid contamination with the clinician’s own microbiota. The vulva was cleaned with water followed by a wipe with 70% ethanol to avoid dragging bacteria into the vagina. The swabs were moistened with sterile phosphate buffered saline (PBS) and taken with the help from a sterile vaginoscope to avoid contamination from the vulva. The swab was collected deep in the vagina, close to the vagino-cervical transition, placed in 1 mL sterile PBS and stored at −80 °C until further processing. As all samples were collected, the swabs were thawed and vortexed with five small autoclaved glass beads for 5 min to release the swab material from the swab.

During sampling on days 0, 5, 9, and 13, the minipigs were anesthetized with an intramuscular (IM) injection of azaperone (40 mg/mL) 2 mL/10 kg together with atropine (1 mg/mL) 0.05 mL/kg, followed by an IM injection of Zoletil^®^ 50 Vet mixture (1 vial Zoletil 50^®^ Vet (125 mg tiletamin and 125 mg zolazepam), 6.25 mL xylazine (20 mg/mL), 1.5 mL ketamine (100 mg/mL) and 2.5 mL methadone (10 mg/mL)) 1 mL/10 kg. Due to observed depressive effects on respiration premedication with azaperone/atropine mixture was suspended on days 17 and 21.

### DNA extraction/purification

Swab material (800 µL) was transferred to a Pathogen Lysis tube S (cat. No 19091, Qiagen, Copenhagen, Denmark) for mechanical disruption and lysis of the wall of Gram positive bacteria. The tubes were processed according to the manufacturer’s instructions. At the end of the protocol, 400 µL of the supernatant was transferred into a new 2 mL microcentrifuge tube and DNA was extracted from this portion with the QIAamp UCP Pathogen Mini Kit (cat no. 50214, Qiagen) according to the manufacturer’s instructions. In the final step DNA was eluted with two times 50 µL AVE buffer (QIAamp UCP Pathogen Mini Kit) into the same elution tube. DNA concentration was measured on NanoDrop 2000c (Thermo Scientific, Copenhagen, Denmark).

### PCR amplification

A 16 s rRNA gene sequence library was constructed with a limited cycle polymerase chain reaction (PCR) amplifying the V3 and V4 region of the 16 s rRNA gene according to the manual from Illumina [22]. Specific primers, selected based on Klindworth et al. [23] to the region of interest with overhang adapters attached were used with the following sequence:

- 16S Amplicon PCR Forward Primer = 5′TCGTCGGCAGCGTCAGATGTGTATAAGAGACAGCCTACGGGNGGCWGCAG
- 16S Amplicon PCR Reverse Primer = 5′ GTCTCGTGGGCTCGGAGATGTGTATAAGAGACAGGACTACHVGGGTATCTAATCC

The PCR was run under the following settings: 95 °C for 3 min, 25 cycles of 95 °C for 30 s, 55 °C for 30 s, 72 °C for 30 s and finally 5 min at 72 °C, where after the samples were stored at 4 °C. The size of the PCR product was expected to be ~550 base pairs (bp) and verified on a Bioanalyzer DNA 1000 chip (Agilent Technology, Santa Clara, CA, USA).

The 16 s V3 and V4 amplicons were purified on AMPure XP Beads (Beckman Culter, Copenhagen, Denmark) according to the manufacturer’s instructions. Illumina sequencing adapters and dual-index barcodes were added to the amplicon target so that the different sample libraries could be pooled for sequencing. It was performed with the Nextera XT Index Kit (Illumina) and a PCR: 95 °C for 3 min, eight cycles of 95 °C for 30 s, 55 °C for 30 s, 72 °C for 30 s and 5 min at 72 °C. Samples were subsequently stored at 4 °C. The final PCR clean-up was performed with AMPure XP beads (Beckman Coulter, Copenhagen, Denmark) and confirmation of the right size of the target (now 630 bp) was performed on a Bioanalyzer DNA 1000 chip (Agilent Technology, CA, USA).

The final step before sequencing was determination of DNA concentration with Qubit (Life Technologies, Carlsbad, CA, USA) and pooling of libraries for one MiSeq run.

### 16S sequencing

The pooled libraries were denatured with NaOH, diluted with hybridization buffer and further heat denatured before running the sequencing. 5% PhiX was included for low diversity libraries. The sequencing was performed with an Illumina MiSeq instrument using paired end 2 × 300-bp reads and a MiSeq v3 reagent kit.

### Sequence processing

The raw reads were separated into different samples according to the sample-specific barcodes/indexes. An initial quality control was performed and the dataset was extracted as read 1 and read 2 FASTQ files on the Illumina MiSeq instrument using the MiSeq reporter according to the manufacturers instructions.

Meta Genome Rapid Annotation using Subsystem Technology (MG-RAST) [24] and QIIME [25] was used to analyze the sequence dataset and determine the taxonomic classification of the microbiome. Overlapping paired-end reads, representing the reads of one sample from the same sequencing run were joined using FastqJoin *script join_paired_ends.py* in QIIME [25]. Pairs with a minimum overlap setting of 6 bp and a maximum difference of 8% were merged into a single file. An average of 32 ± 15% (SD) of the initial sequence reads from each sample was removed in this step. The final sequence length was 452 ± 6 bp (Mean ± SD) and average number of joined reads per sample was approximately 107 373.

A metadata file describing the origin of all samples and providing information about sequencing was uploaded and validated by MG-RAST. Joined sequence reads were submitted to MG-RAST using the default quality control filtering parameters of the MG-RAST program. The filtering step removed between 0.3 and 2.6% sequences in each sample.

### Sequence analysis

All reads that passed MG-RAST quality control were searched against a reduced RNA database to identify ribosomal RNA using Blast-Like-Alignment Tool (BLAT) at a minimum of 90% identity. In average 96.0 ± 6.7% of the reads were identified as ribosomal RNA. The detected rRNA-similar reads were clustered at 97% identity, and the longest sequence was chosen as the cluster representative. BLAT similarity search for the longest cluster representative was performed against the M5rna database [26], which integrates RNA databases implemented in MG-RAST (namely SILVA, Greengenes, and RDP).

Taxonomy assignment was based on best-hit classification using the M5NR database [27] with a maximum e-value cutoff of 10^−5^, minimum identity cutoff of 60%, and minimum alignment cutoff of 15%. The relative abundance of each species was calculated based on the number of hits for that species. MG-RAST—generated taxonomy abundance was further normalized by number of reads mapped to each sample. Rarefaction curves and PCoA plots were made with MG-RAST.

BIOM table and metadata suitable for data analysis with QIIME were created using the MG-RAST server. The table contained information on all taxa abundance and the number of hits (or clusters) found for the taxon in each sample. The abundance was calculated by multiplying the actual number of database hits found for the clusters by the number of cluster members, and was used to make various charts in QIIME. Before the analysis, positive filtering using QIIME script *filter_taxa_from_otu_table.py* was applied to process BIOM table in a way that only bacteria taxa were retained.

To calculate species diversity within individual samples alfa diversity was calculated using the *alpha_diversity.py* script. Furthermore, the Simpson index was calculated taking species abundance into account. Bray-Curtis non-phylogenetic based metrics (β-diversity) was used to compare species diversity between the samples. The following scripts *beta_diversity.py*, *principal_coordinates*, *make_2d_plots.py*, *make_emperor.py* were used to create 2D PCoA plots.

Finally, the core microbiome (operational taxonomic units (OTUs) present in 90% of the samples) in samples grouped based on the life stage (P, M_0-M_21) from the BIOM table was calculated and the distribution of bacteria at phylum, class, order and family was summarized and plotted using the script *summarize_taxa_through_plots.py*.

The complete sequence dataset was deposited on the MG-RAST server as Vaginal Bacterial Flora in Gottingen Minipigs [28].

### Negative controls/quality assurance

After thawing the samples, all procedures were performed in a LAF bench and the DNA extraction was performed with QIAamp UCP Pathogen Mini Kit (cat no. 50214, Qiagen). To make sure that no bacterial contamination was introduced with the swabs or during the DNA extraction, the following negative controls were included: one swab was unpacked and put directly into a sample tube similar to the other samples with 0.8 mL PBS and one control with just PBS were run through the DNA extraction kit and DNA content was measured on a NanoDrop instrument together with the AVE buffer from kit. Furthermore, a negative control was included in the V3 + V4 16S region PCR amplification assay to make sure that the PCR reagents were not a source of contamination.

### Statistics

All statistical analyses were performed in GraphPad Prism 5 (GraphPad Software Inc., CA, USA). Gaussian distribution of data was analysed by D’Agostino and Pearson omnibus normality test in Graph Pad Prism. Normally distributed data/multiple groups were analysed with ANOVA and if a significant difference was identified, multiple comparisons were performed with Bonferroni comparison test.

The Pearson test was used to analyse if the normalised and ranked abundance of the OTUs at family level were significantly correlated between the different groups/timepoints. The comparisons were considered statistical significant if the *p* value was lower than 0.05 (*p* < 0.05). Further levels of significance is indicated with asterisks *P < 0.05, ** *P* < 0.01, *** *P* < 0.001.

## Results

The vaginal microbiota was characterized in ten prepubertal minipigs and ten sexually mature minipigs during an estrous cycle by 16S rRNA gene sequencing. Genomic DNA was isolated from vaginal swab samples and the V3 + V4 regions of the 16S rRNA gene was amplified and sequenced on an Illumina MiSeq instrument. Negative controls were included and no DNA was detected in any of the three controls. After quality control of the resulting sequences, the resulting dataset consisted of 7 516 123 high-quality sequences that were uploaded to MGRAST. The average length was 446 bp and the mean number of reads per sample was 107 373. Sequence analysis and taxonomic assignment of the reads were performed in MG-RAST and QIIME. Rare sequences were removed and only sequences present in 90% of the samples were included in the core microbiome.

**Figure 2:**
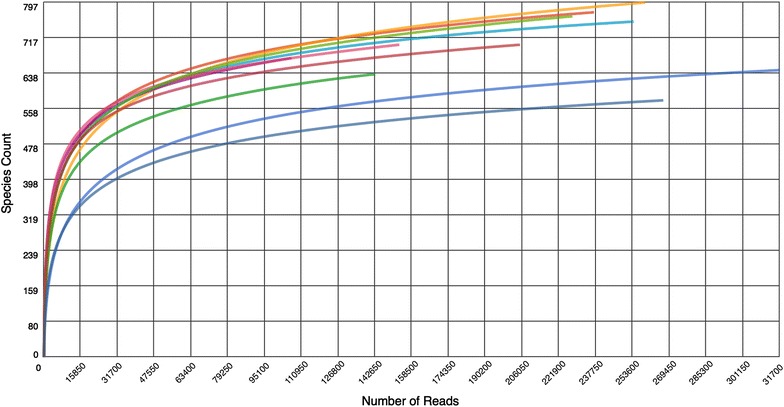
**Rarefaction curves for the prepubertal minipigs.** Rarefaction curves were made for all samples to evaluate the species richness, depth of sampling and sequencing coverage. 97% (68/70) of the curves reached a clear plateau stage examplified with the ten prepubertal samples. The clear plateu stage indicates that the depth of the sampling and sequencing was sufficiently to detect a nearly maximum number of species.

Rarefaction curves were made to evaluate the species richness in each sample and to evaluate if the depth of the sequencing was sufficient. Rarefaction curves for 97% of the individual samples approached a plateau stage, indicating that the sampling of the community and sequencing coverage was sufficiently deep to detect a nearly maximum numbers of OTUs. Examples of rarefaction curves for the prepubertal minipigs are shown in Figure 2.

### The vaginal microbiota in prepubertal minipigs

**Figure 3:**
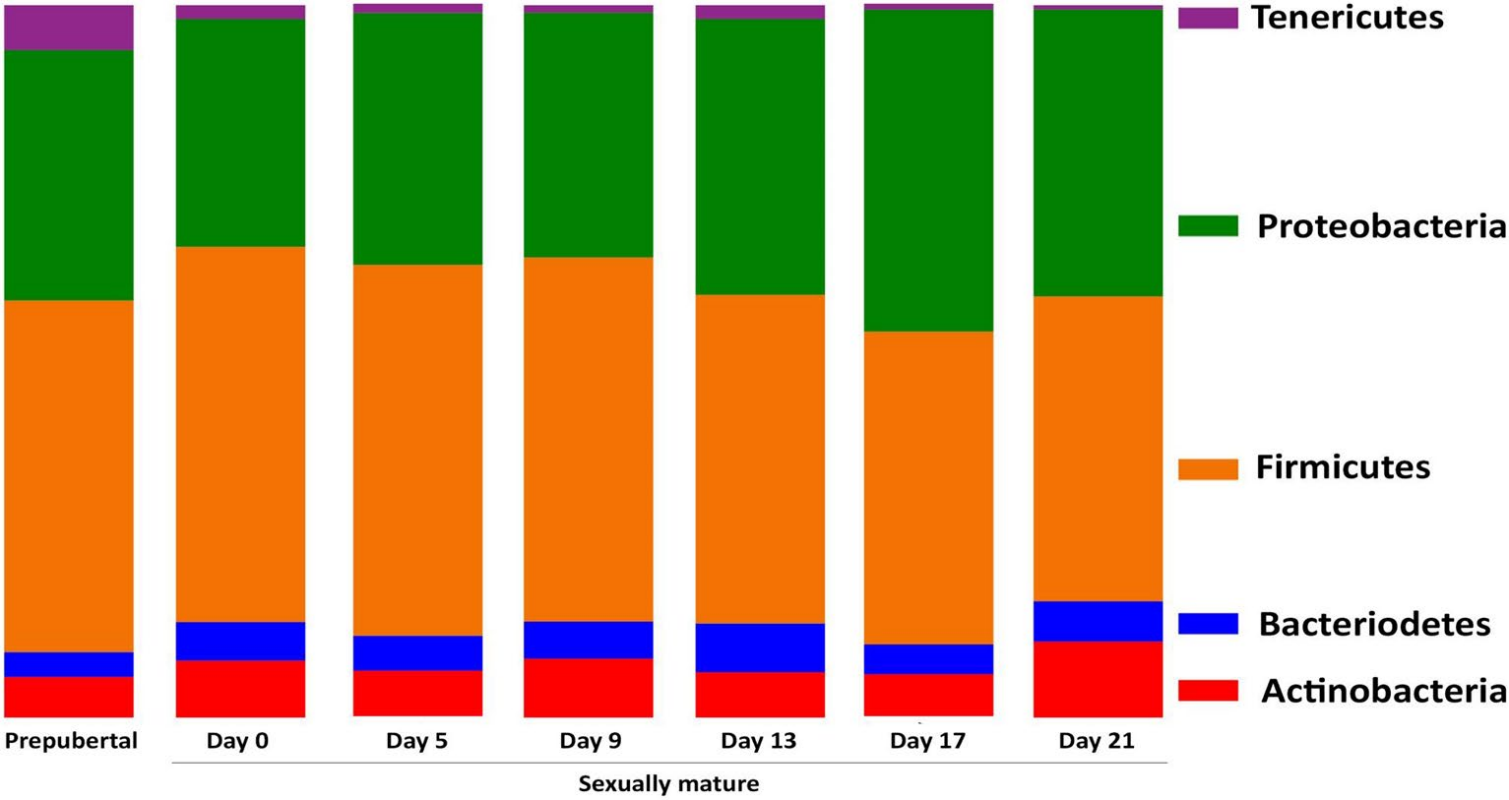
**Vaginal core microbiome compositions at phylum level in prepubertal and sexually mature minipigs.** DNA was extracted from vaginal swab samples and 16 s rRNA gene sequencing (300 bp paired end) was performed on an Illumina MiSeq platform. The resulting sequences that passed quality assurance were assigned to bacterial sequences in the M5rna database with MG-RAST. The core microbiome (those OTUs that were present in 90% of the samples) was analysed and the relative abundance of OTUs was calculated in the different samples. Each column represents the mean abundance in the ten minipigs assigned to each group. The vaginal core microbiome is dominated by *Firmicutes* in both prepubertal and sexually mature minipigs, followed by *Proteobacteria*, *Actinobacteria* and *Bacteriodetes.*

**Figure 4:**
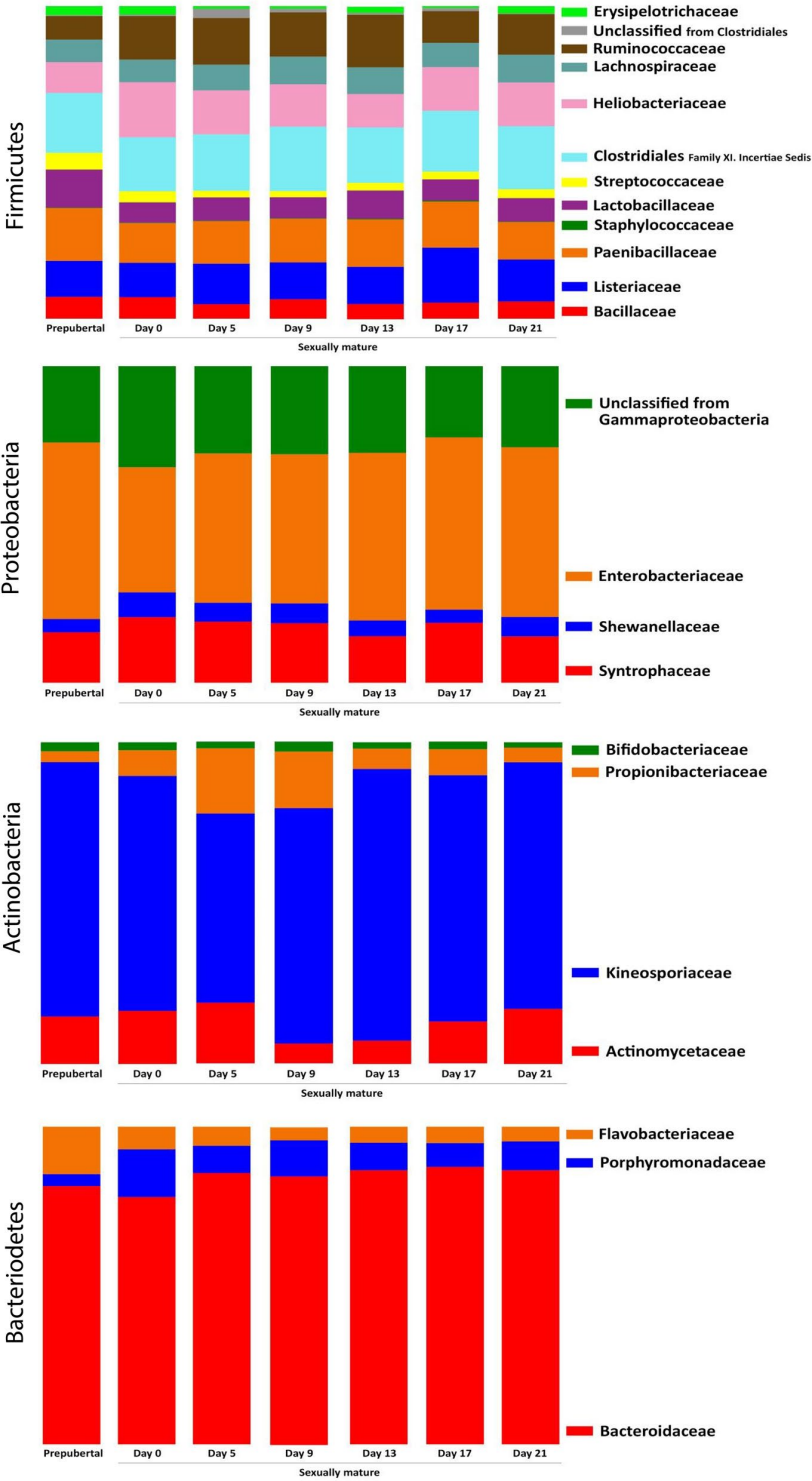
**Composition of families within the four dominating phyla in the vaginal core microbiome in minipigs.** The relative abundance of families in the core microbiome within the four dominating phyla. Each column represent the mean abundance (*n* = 10). *Firmicutes* constitutes the greatest diversity.

In the prepubertal Göttingen Minipigs, the core microbiome was established by five phyla: Firmicutes (49.3%), Proteobacteria (35.2%), Tenericutes (6.3%), Actinobacteria (5.7%) and Bacteriodetes (3.5%) (Figure 3). The composition of families within the four dominating phyla is shown in Figure 4 and the overall composition of bacterial families is shown in Table 1. When looking at the individual samples at family level (Additional file 1) the inter-individual standard deviation (SD) in the abundance ranged from 0.03–6.4% within *Fusobacteriaceae* and *Enterobacteriaceae* respectively. Hence the largest inter-individual variation was seen within the *Enterobacteriaceae*. The median SD was 0.61%. All SD values are given in Additional file 1.

**Table 1:** **Distribution of family OTUs in the vaginal microbiota in prepubertal and sexually mature Göttingen Minipigs.**

Prepubertal (*n* = 10), mean abundance%		Sexually mature (*n* = 10), mean abundance%, range over the different days in the estrous cycle (min–max%)	
19.6%	*Enterobactericeae*	18.7% (12.6–24.6%)	*Enterobacteriaceae*
9.5%	*Clostridiales* family XI incertae sedis	10.1% (9.6–10.6%)	Unclassified assigned to *Gammaproteobacteria*
8.5%	Unclassified assigned to *Gammaprotebacteria*	9.1% (8.2–10.5%)	*Clostridiales* family XI incertae sedis
8.4%	*Paenibacillaceae*	6.8% (4.9–9.3%)	*Heliobacteriaceae*
6.3%	*Entomoplasmataceae*	6.7% (5.7–8.6%)	*Syntrophaceae*
6%	*Lactobacillaceae*	6.7% (4.4–0.8%)	*Ruminococcaceae*
5.7%	*Listeriaceae*	6.6% (5.1–7.2%)	*Paenibacillaceae*
5.6%	*Syntrophaceae*	6.2% (5.5–7.7%)	*Listeriaceae*
4.9%	*Heliobacteriaceae*	5.6% (3.8–8.2%)	*Kineosporiaceae*
4.5%	*Kineosporiaceae*	4.5% (3.6–5.9%)	*Bacteriodaceae*
3.7%	*Ruminococcaceae*	4% (3.4–4.5%)	*Lachnospiraceae*
3.6%	*Lachnospiraceae*	3.5% (3–4.2%)	*Lactobacillaceae*
*3.5%*	*Bacillaceae*	2.7% (2.3–3.6%)	*Bacillaceae*
2.8%	*Bacteriodaceae*	2.2% (1.9–2.5%)	*Shewanellaceae*
2.6%	*Streptococcaceae*	1.3% (0.6–2%)	*Entomoplasmataceae*
1.5%	*Shewanellaceae*	1.2% (1–1.9%)	*Streptococcaceae*
1.4%	*Erysipelotrichaceae*	1% (0.5–1.8%)	*Actinomycetaceae*
0.8%	*Actinomycetaceae*	0.8% (0.4–1.5%)	*Propionibacteriaceae*
0.5%	*Flavobacteriaceae*	0.7% (0.3–1.4%)	*Erysipelotrichaceae*
0.2%	Unclassified from *Clostridiales*	0.6% (0.1–1.6%)	Unclassified from *Clostridiales*
0.2%	*Propionibacteriaceae*	0.5% (0.3–0.8%)	*Porphyromonadaceae*
0.2%	*Bifidobacteriaceae*	0.3% (0.2–0.4%)	*Flavobacteriaceae*
0.1%	*Porphyromonadaceae*	0.2% (0.1–0.3%)	*Bifidobacteriaceae*
0.1%	*Staphylococcaceae*	0.1% (0.1–0.1%)	*Staphylococcaceae*

On genus level, the most common OTU was unclassified and derived from *Clostridiales Family XI. Incertae Sedis* (9.5%), followed by an unclassified OTU derived from *Gammaproteobacteria* (8.5%), *Paenibacillus* (8.4%), *Mesoplasma* (6.3%), *Proteus* (6.3%), *Lactobacillus* (6%), *Listeria* (5.7%) and the remaining genera can be seen in Additional file 2.

### The vaginal microbiota in sexually mature minipigs during an estrous cycle

The sexually mature Göttingen Minipigs were hormonally synchronized and sampled on six time points during the estrous cycle to investigate if systematic fluctuations occurred in the composition of the vaginal microbiome. On all sampling days during the estrous cycle in the sexually mature Göttingen Minipigs, the same five phyla constituted the core microbiome: Firmicutes with an average abundance of 48.1% (range 42.8–52.7%), Proteobacteria (37.7%, range 32–40.3%), Actinobacteria (7.6%, range 5.9–10.7%), Bacteriodes (5.4%, range 4.2–6.8%) and Tenericutes (1.3%, range 0.6–2%) (Figure 3).

The composition of different bacterial families within the four dominating phyla is shown in Figure 4. No statistical significant fluctuation was found in the composition of bacterial families on the different days of the estrous cycle and the overall composition of bacterial families is therefore given as the average abundance with the range in Table 1. When looking at the individual samples across all sampling days at family level (Additional file 1) the inter-individual SD on the abundance was 0.02–14.9% within *Corynebacteriaceae* and *Enterobacteriaceae* respectively, and the median SD was 0.8% (Additional file 1). Hence the largest inter-individual variation across sampling days was seen within the *Enterobacteriaceae.* All SD values are given in Additional file 1.

On genus level, the most common OTU was unclassified and derived from *Gammaproteobacteria* followed by an unclassified OTU derived from *Clostridiales Family XI. Incertae Sedis*, *Yersinia*, *Paenibacillus*, *Syntrophus*, *Heliobacterium*, *Listeria*, *Faecalibacterium*, *Proteus* and the remaining genera can be seen in Additional file 2.

### Comparison of prepubertal and sexually mature minipigs

**Figure 5:**
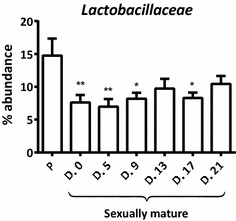
**The abundance of** ***Lactobacillaceae*** **in prepubertal and sexually mature minipigs during an estrous cycle.** When evaluating the abundance of each family within prepubertal and sexually mature minipigs, we found a higher abundance of *Lactobacillaceae* in the prepubertal minipigs than in the sexually mature. The difference between the prepubertal and sexually mature minipigs was significant on day 0, 5, 9 and 17 of the estrous cycle. The abundance is given as percentage of *Lactobacillaceae* within the *Firmicutes* phylum. The bars represent mean + SEM (*n* = 10). Statistical analysis was performed with multiple bonferroni-corrected comparisons. Asterisks indicate significance levels: * *p* < 0.05, ** *p* < 0.01.

When evaluating the abundance of each family in prepubertal and sexually mature Göttingen Minipigs, we found a higher abundance of *Lactobacillaceae* within the *Firmicutes* phylum in the prepubertal minipigs than in the sexually mature ones during an estrous cycle (Figure 5). No significant differences were found in the abundance of the remaining families.

### Diversity

The diversity in each sample was calculated with the α-diversity and Simpson index (Table 2). α-diversity is a parameter for within-community diversity and the Simpson index combines species richness and abundance to evaluate the even distribution of OTUs in the samples [29]. The different parameters are shown in Table 2. We found no significant differences in the diversity and evenness parameters among the different groups, hence the species richness and distribution of OTUs appears to be similar in prepubertal and sexually mature minipigs regardsless of stage in estrous cycle.

**Table 2:** **α-diversity and Simpson’s diversity index in prepubertal and sexually mature minipigs.**

	Prepubertal	Sexually mature (day in estrous cycle)					
		Day 0	Day 5	Day 9	Day 13	Day 17	Day 21
α-diversity	56.5 ± 30.6	90.3 ± 23.1	47.5 ± 28.1	66.7 ± 29.4	58.5 ± 28.1	53.5 ± 39.5	57.5 ± 36.6
Simpson	0.95 ± 0.007	0.95 ± 0.007	0.94 ± 0.02	0.94 ± 0.01	0.94 ± 0.009	0.93 ± 0.04	0.94 ± 0.01

Diversity indexes were calculated to determine if the species richness and abundance differed in the prepubertal and sexually mature minipigs during an estrous cycle. The shown values represent mean ± SD. We found no significant difference between the prepubertal and sexually mature minipigs on different time points during an estrous cycle. It states that the species diversity, richness and abundance/distribution of species is similar in prepubertal and sexually mature minipigs during an estrous cycle (ANOVA results for α-diversity *p* = 0.079 and for the Simpson index *p* = 0.11).

**Figure 6:**
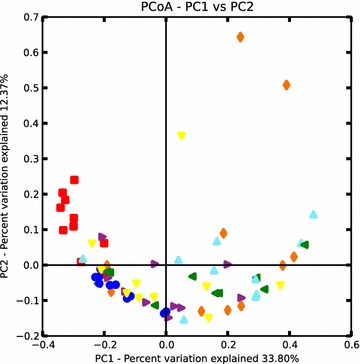
**The principal coordinate analysis on the vaginal microbiome in prepubertal and sexually mature minipigs.** Principal coordinate analysis (PCoA) based on Bray Curtis β-diversity showed no clear distinct clustering of the prepubertal (red) and sexually mature minipigs on the different days of the estrous cycle (day 0 = blue, day 5 = orange, day 9 = green, day 13 = purple, day 17 = yellow, day 21 = light blue).

Based on the β diversity/Bray-Curtis non-phylogenetic based metrics a principal coordinate analysis (PCoA) was performed to evaluate diversity between the communities and groups. The PCoA plot (Figure 6) showed no significant separation or distinct clustering, which confirms absence of significant differences in the taxonomic composition of the vaginal microbiome in the examined Göttingen Minipigs.

**Figure 7:**
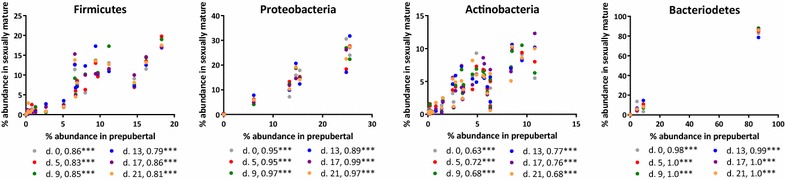
**Correlation of family composition within the dominating phyla in prepubertal and sexually mature minipigs.** To evaluate if the composition of families within the four dominating phyla was similar in the prepubertal minipigs and the sexually mature minipigs on the different days during the estrous cycle a Pearson’s correlation test was performed. It showed a strong correlation between the distribution of families in the prepurtal minipigs and the distribution of families in the sexually mature minipigs on the different days during the estrous cycle. The R squared is given for each day with superscript *p* value summary, *** equals a *p* < 0.001.

This was further confirmed in the Pearson’s test, investigating if the ranked normalised abundance of the OTUs in the prepubertal Göttingen Minipigs were significantly correlated to all time points in the sexually mature minipigs (Figure 7). We found a strong significant correlation (*p* < 0.001) in the distribution of taxonomic families within the four phyla between the two groups of minipigs (Figure 7).

## Discussion

The introduction of the female Göttingen Minipig as a model of genital tract infections in women, and the important role of the vaginal microbiota for a healthy vaginal environment, have created a need for increased knowledge on the minipig vaginal microbiota [11, 30, 31]. This study revealed that the vaginal microbiome in Göttingen Minipigs was composed of *Firmicutes* (60%), *Proteobacteria* (20.6%), *Actinobacteria* (14.6%), *Fusobacteria* (3.6%), and *Bacteroidetes* (1.2%). This is quite comparable to the vaginal microbiota in women, which is dominated by *Firmicutes* and further composed of *Bacteriodetes*, *Actinobacteria* and *Proteobacteria* [32]. However, in women, lactobacilli are the dominating species, making up 70–100% of the microbiota, in a healthy vaginal environment [31–33]. This is apparently not the case in Göttingen Minipigs where *Lactobacillaceae* represented 6% of the microbiota in prepubertal minipigs and in average 3.5% in the sexually mature minipigs. The higher abundance of *Lactobacillaceae* in the prepubertal minipigs compared to the sexually mature ones differs from the findings in women. In prepubertal women around 38% of the aerobic microbiota are lactobacilli when determined by cultivation [34]. Even though, different methods have been used (aerobic cultivation in prepubertal women and culture independent studies in adults) and slight different sites have been sampled (mid-vagina/posterior fornix) and a direct comparison therefore is difficult, it seems as if *Lactobacillus* spp. become more numerous after puberty in women [31–34]. The opposite pattern, found in this study, with a higher abundance of *Lactobacillaceae* in the prepubertal minipigs might be influenced by different environmental conditions such as housing, bedding, feeding and handling etc. However, both the prepubertal and sexually mature minipigs were fed a minipig SDS diet, autoclaved grass straw and the bedding material and water were tested free of relevant contaminants such *E. coli* and other *Enterobacteriaceae*. Hence the variation in environmental influence should be minimal in this study.

Earlier studies on the porcine vaginal microbiota found *Streptococcus* spp., *E. coli*, *Staphylococcus* spp., *Corynebacterium* spp., *Micrococcus* spp. and *Actinobacillus* spp. as the dominating species by cultivation [17, 18]. These species were also detected in this study, but only with small abundances and not as dominating species/OTUs. *Streptococcus* spp. constituted in average 1.4% of the vaginal flora, *E. coli* 3.7%, and *Staphylococcus* spp. 0.4%. This illustrates the great influence of methods on the obtained results and that culture studies have significant limitations. Detection of microbiota by culturing will only show the culturable population of the microbiota, hence a selected (very biased) part of the microbiota, since non-culturable bacteria will not be detected by this method [16, 19, 35]. However, a disadvantage with the 16 s rRNA gene sequencing is that this method will detect DNA from both alive and dead bacteria, whereas cultivation only detects the living cultivable bacteria [35–37]. Sequencing on RNA could be performed to detect metabolically active bacteria [37].

In humans, the vaginal microbiota is initially established during the birth, from the microbiota of the mother’s vagina and with bacteria from the skin and surrounding environment [38]. The difference in the vaginal microbiota between women and pigs, mainly in the proportion of lactobacilli, may be explained by environmental factors. Many of the family OTUs identified in this study have also been detected in the fecal microtioba of Göttingen and Ossabaw minipigs such as *Prevotellaceae, Clostridiaceae, Lachnospiraceae, Bacteriodaceae Ruminococcaceae, Enterobacteriaceae, Streptococcaceae,* and *Lactobacillaceae* [39, 40] and the porcine composition of the fecal microbiota is indeed very similar to the vaginal microbiota [39, 40]. The close similarity to the minipig fecal microbiota indicates that a major part of the minipig vaginal microbiota is likely to be derived from fecal transmission. However, since the microbiota was only determined at the family level, it is possible that the individual species and strain composition within each family differ between the feces and the vaginal mucosa. In women, it has been confirmed at strain level that a part of the vaginal microbiota is likely to originate from the rectal microbiota [41, 42]. A study [41] found that 44% of the isolated strains from the vaginal microbiota were common for both the vagina and the rectum. In women, it is not known if it is bacteria from the vagina that colonize rectum or vice versa [41]. In pigs, we hypothesize that it is the rectal microbiota that most likely enters the vaginal environment due to the anatomical location of the rectum just dorsal to the vulva. Furthermore, pigs are exposed to a higher degree of fecal contamination than women.

The analysis of species richness and species evenness revealed a similar composition of the microbiota in prepubertal minipigs and sexually mature minipigs. However, even though the microbiota seems stable at the family level there can still be variation at the genus and species levels within each family. In women, the composition of the vaginal microbiome can be highly fluctuating both between individuals and during the menstrual cycle [15, 16, 43]. Parameters such as diet, intime hygiene, changing sexual partners and especially menstruation are known to cause undesired fluctuations in the microbiota in women [30]. Hence the minipig show limitations in resembling these circumstances in women, however, the minipig offers a model system with a stable microbiota for the study of the impact of isolated parameters without the undesired fluctuations in the microbiota.

Some of the limitations of this study were that we only included one sampling site (the anterior vagina) and only looked at bacterial organisms. In women, sampling sites such as the cervix and the outer vagina differ slightly in the composition of the microbiota [44]. This was not evaluated in this study and the microbiota in the porcine vestibulum (*vestibulum vaginae*) might differ from what we have found in this study. Furthermore, viruses and fungi also play an important role in the vaginal microenvironment [45], but they were not evaluated in this study.

In terms of resolution of the sequencing data, it was not possible to assign all the sequences to known bacteria with the used primers and sequencing protocol. The unassigned OTUs at species level are likely to be new yet undescribed bacteria although methodical problems such as base calling errors may also explain some of the novel sequences [46].

In conclusion, this study showed that the vaginal microbiota in Göttingen Minipigs was dominated by the phyla *Firmicutes, Proteobacteria, Actinobacteria*, *Bacteriodetes* and *Tenericutes*. The dominating OTU was not lactobacilli as seen in women, but unassigned species belonging to *Gammaproteobacteria*, *Enterobacteriaceae* and *Clostridiales*. The unassigned bacteria are likely to be yet undescribed species and further analysis are needed to characterize these species. Based on PCoA plots and further statistical analysis, we found no significant difference in the composition of the microbiota on family level before and after puberty or during an estrous cycle in sexually mature Göttingen Minipigs. We found a higher abundance of *Lactobacillaceae* in the prepubertal minipigs compared to the sexually mature minipigs, but otherwise the composition of the vaginal microbiota was very stable between individual minipigs, before and after puberty, and during an estrous cycle. The stable and now characterized vaginal microbiota adds important knowledge to be considered when using the Göttingen Minipig as a model of genital tract infections in women and the porcine microbiota in general.

## Additional files

10.1186/s13567-015-0274-0 **Abundance of OTUs at family level in individual samples in prepubertal and sexually mature minipigs.** This file shows the distribution of bacterial families in the individual samples in prepubertal and sexually mature minipigs to visualize the inter-individual variation. The first spreadsheet contains the data from the prepubertal minipigs and the second spreadsheet contains data from the sexually mature minipigs.
10.1186/s13567-015-0274-0 **Abundance of OTUs at genus level in prepubertal and sexually mature minipigs**. This file shows the abundance of genera in prepubertal and sexually mature minipigs. The first spreadsheet contains the data from the prepubertal minipigs and the second spreadsheet contains data from the sexually mature minipigs.

## Abbreviations

Bp: base pairs
LAF: laminar air flow
OUT: operational taxonomic units
PBS: phosphate-buffered saline
PCoA: principal coordinates analysis
PCR: polymerase chain reaction
rRNA: ribosomal ribonucleuic acid
Spp.: species
STI: sexually transmitted infection
UCP: ultraclean production
